# Human milk miRNAs primarily originate from the mammary gland resulting in unique miRNA profiles of fractionated milk

**DOI:** 10.1038/srep20680

**Published:** 2016-02-08

**Authors:** Mohammed Alsaweed, Ching Tat Lai, Peter E. Hartmann, Donna T. Geddes, Foteini Kakulas

**Affiliations:** 1 School of Chemistry and Biochemistry, The University of Western Australia, Crawley, Western Australia, Australia; 2 College of Applied Medical Sciences, Majmaah University, Almajmaah, Riyadh, Saudi Arabia

## Abstract

Human milk (HM) contains regulatory biomolecules including miRNAs, the origin and functional significance of which are still undetermined. We used TaqMan OpenArrays to profile 681 mature miRNAs in HM cells and fat, and compared them with maternal peripheral blood mononuclear cells (PBMCs) and plasma, and bovine and soy infant formulae. HM cells and PBMCs (292 and 345 miRNAs, respectively) had higher miRNA content than HM fat and plasma (242 and 219 miRNAs, respectively) (*p* < 0.05). A strong association in miRNA profiles was found between HM cells and fat, whilst PBMCs and plasma were distinctly different to HM, displaying marked inter-individual variation. Considering the dominance of epithelial cells in mature milk of healthy women, these results suggest that HM miRNAs primarily originate from the mammary epithelium, whilst the maternal circulation may have a smaller contribution. Our findings demonstrate that unlike infant formulae, which contained very few human miRNA, HM is a rich source of lactation-specific miRNA, which could be used as biomarkers of the performance and health status of the lactating mammary gland. Given the recently identified stability, uptake and functionality of food- and milk-derived miRNA *in vivo*, HM miRNA are likely to contribute to infant protection and development.

Human milk (HM) is the optimal nutrition for term infants^1^. In addition to being a food source, HM confers developmental programming to the infant and protection against infections, resulting in decreased risk of sudden infant death syndrome and reduced mortality and morbidity both in the short- and long-term^2^,^3^,^4^,^5^,^6^. These effects are mediated by HM-specific regulatory factors including both cellular and biochemical components^7^,^8^,^9^,^10^. In contrast, artificial infant formula cannot confer such protective and developmental functions as it lacks important HM components with bioactivity^11^,^12^. An additional unique bioactive component of HM that has been recently discovered is miRNAs^13^,^14^.

miRNAs are small non-coding RNAs, which regulate gene expression, thus control protein synthesis at the post-transcriptional level in eukaryotic cells^15^. They have been identified as key regulators of diverse biological and developmental processes in eukaryotes (cell proliferation and differentiation, apoptosis, immune system development and immune response^16^,^17^) by targeting messenger RNA (mRNA) during its translation into protein, either degrading the mRNA or inhibiting the translation process^18^. Aberrant miRNA expression has been found to be associated with pathologies, including different types of cancer, inflammation and diabetes^19^. Importantly, food-derived miRNA have been recently shown to be very stable in the gastrointestinal tract and be transferred to the blood circulation of adults, influencing gene expression in different tissues^20^. In addition to tissues and cells, miRNAs have been isolated from body fluids, such as plasma, urine, saliva and tears^14^. Further, exosomes, small cell-derived vesicles present in body fluids and carrying proteins and molecules, have been shown to take up miRNAs mediating their protection against digestion and facilitating their regulatory functions in different tissues and organs^21^.

Most recently, miRNAs have been isolated in high quantities from both animal and HM, and were shown to be present both as free molecules in skim milk^13^,^14^,^22^,^23^,^24^ and packaged in vesicles such as milk exosomes and the fat globule^25^,^26^,^27^,^28^. Bovine milk exosomes can be transported by intestinal cells via endocytosis in the human and rat colon^29^. Moreover, studies of bovine milk consumed by adult humans showed that at least some bovine milk miRNA can be transferred to the bloodstream^30^. This was further reinforced by Arntz *et al.*^31^, who demonstrated *in vitro* uptake by splenocytes and intestinal cells of miRNA derived from bovine milk, and their therapeutic role in delaying the onset of experimental arthritis when delivered orally to mice. Collectively, these findings strongly suggest that HM miRNA survive the gastrointestinal tract of the infant to exert regulatory functions during breastfeeding^12^, similar to what has been recently shown for maternal milk-derived stem cells^32^. It is therefore important to elucidate the origin, properties, distribution and functional significance of HM miRNA as a novel regulatory component of milk.

Most previous research in milk miRNA has focused on animal milk, including the bovine^22^, porcine^27^ and murine^23^. In these animal studies, next generation sequencing (NGS) has been mainly employed as a miRNA profiling method. Studies using NGS and other global miRNA profiling methods in HM are scarce, with many previous investigations mainly using qPCR-based technology for a limited number of miRNAs. Further, skim milk and to a lesser extent, milk fat have been the milk fractions of choice in previous milk miRNA studies^13^,^14^,^25^,^28^, whilst the milk cellular fraction has been largely neglected despite being a rich source of RNA^33^. Although Munch *et al.* (2013) stated that HM fat was the richest milk fraction in miRNA^25^, this was not compared with HM cells, which potentially conserve high quantities of miRNAs. Recently, we have shown that skim milk has the lowest miRNA content amongst the three HM fractions (cells, fat and skim milk)^34^. Because of this and considering that breastfed infants consume whole HM, it is imperative that further to skim milk, the miRNA content of HM cells and fat is rigorously examined to illuminate the true contribution of HM miRNAs to infant health. At the same time, comparisons with maternal blood may shed light into the origin of HM miRNA, which is still unexplored.

In this study, we profiled 681 HM mature miRNAs using the TaqMan miRNA OpenArray system (Applied Biosystems), with the aim to determine the miRNA composition of the cell and fat fractions of HM and compare it with maternal peripheral blood mononuclear cells (PBMCs) and plasma collected from exclusively breastfeeding women as well as two commercial infant formulae. In addition, gene ontology for miRNA targets and pathway analyses were conducted for the miRNAs that were differentially expressed between HM and maternal blood. Our study elucidates the origin of HM miRNAs and reveals the contributions of the cells and fat fractions to the total content of miRNAs in HM.

## Results

### Concentration and quality of RNA enriched in miRNA in maternal milk and blood

**Figure 1:**
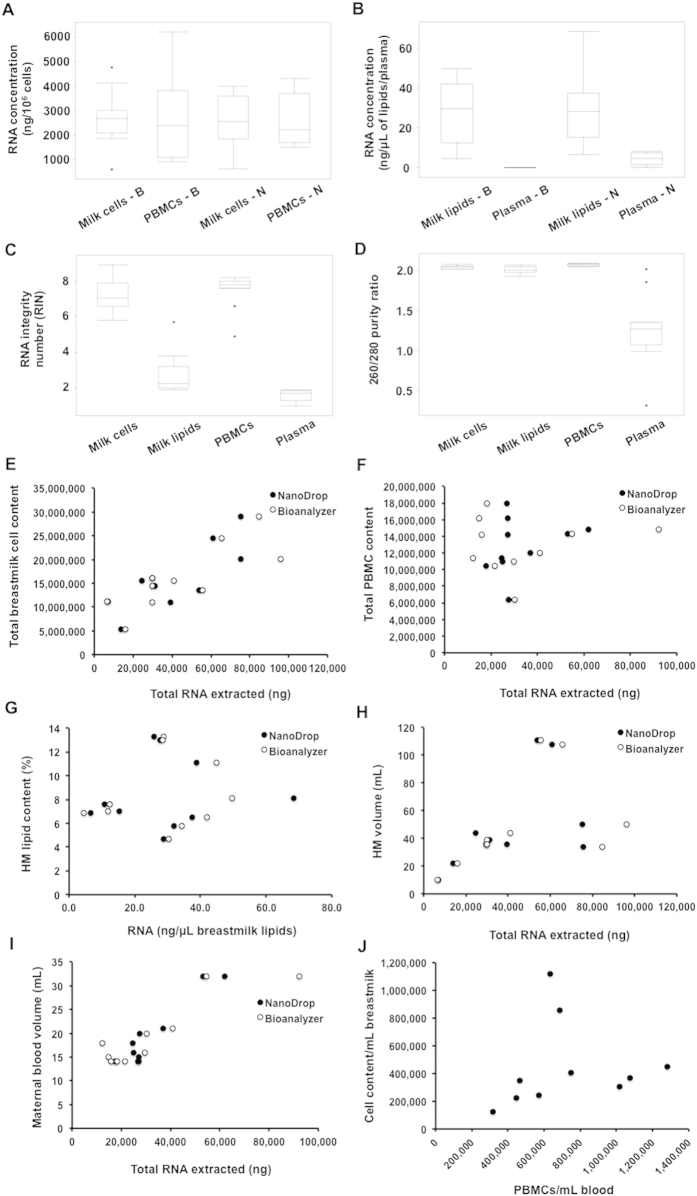
RNA enriched in miRNA in HM cells and fat, and maternal PBMCs and plasma, and associations with HM components. **(A**,**B)** RNA concentration of HM cells, PBMCs, HM fat, and plasma, obtained with NanoDrop 2000 (N) and the Bioanalyzer 2100 (B). **(C,D)** RNA integrity measured by the Bioanalyzer 2100, and RNA purity (260/280 ratio) using NanoDrop 2000 in all four sample groups. **(E,F)** Associations between total RNA eenriched in miRNA and HM cell content or maternal blood PBMC content using Bioanalyzer 2100 and Nanodrop 2000. **(G)** HM fat content (%) and RNA concentration of HM fat (ng). **(H,I)** Associations between HM volume or maternal blood volume with the total RNA enriched in miRNA. **(J)** Association between PBMC content of blood and HM cell content.

Total RNA was extracted using the miRNeasy mini kit for milk cells and PBMCs, miRCURY RNA Isolation-Biofluids Kit for milk fat, and mirVana PARIS Kit for plasma, which have been shown to enrich for miRNA; therefore, the total RNA concentration in the text below refers to total RNA enriched in miRNA. A high correlation between RNA Integrity Number (RIN) using the Bioanalyzer and 260/280 ratio using the NanoDrop was obtained (*p* < 0.001). The total RNA concentration of PBMCs (ng/10^6^ cells) was not different to that of milk cells using either the Bioanalyzer (*p* = 0.960) or the NanoDrop (*p* = 0.800) (Table 1, Fig. 1A). However, both the total RNA concentration and RNA quality of plasma were significantly lower than that of milk fat using both instruments (*p* < 0.001) (Fig. 1B–D). The quality of RNA obtained from milk fat was also lower than that of milk cells and PBMCs when using Bioanalyzer (*p* < 0.001), although still within the acceptable range when using NanoDrop (Fig. 1C,D).

High HM cell content was associated with high total RNA amounts extracted from milk cells (*p* = 0.004 and *p* = 0.005, using Bioanalyzer and NanoDrop, respectively), although the same was not observed for either maternal PBMCs and their total RNA content (*p* = 0.502 and *p* = 0.762, using Bioanalyzer and NanoDrop, respectively) or HM fat content and total RNA/μL fat extracted (*p* = 0.664 and *p* = 0.825 using the Bioanalyser and NanoDrop, respectively) (Fig. 1E–G). A trend for a positive association was seen between the volume of milk expressed and the total RNA extracted from milk cells as well as the maternal blood volume collected and the total RNA extracted from PBMCs, although these were not statistically significant (n = 10) (*p* = 0.172, 0.712 and *p* = 0.482, 0.117 using Bioanalyzer and NanoDrop for HM and blood, respectively) (Fig. 1H,I). Further, no relationship existed between the total number of milk cells and PBMCs (*p* = 0.459) (Fig. 1J).

### Human milk has a diverse miRNA composition that differs from maternal blood

**Figure 2:**
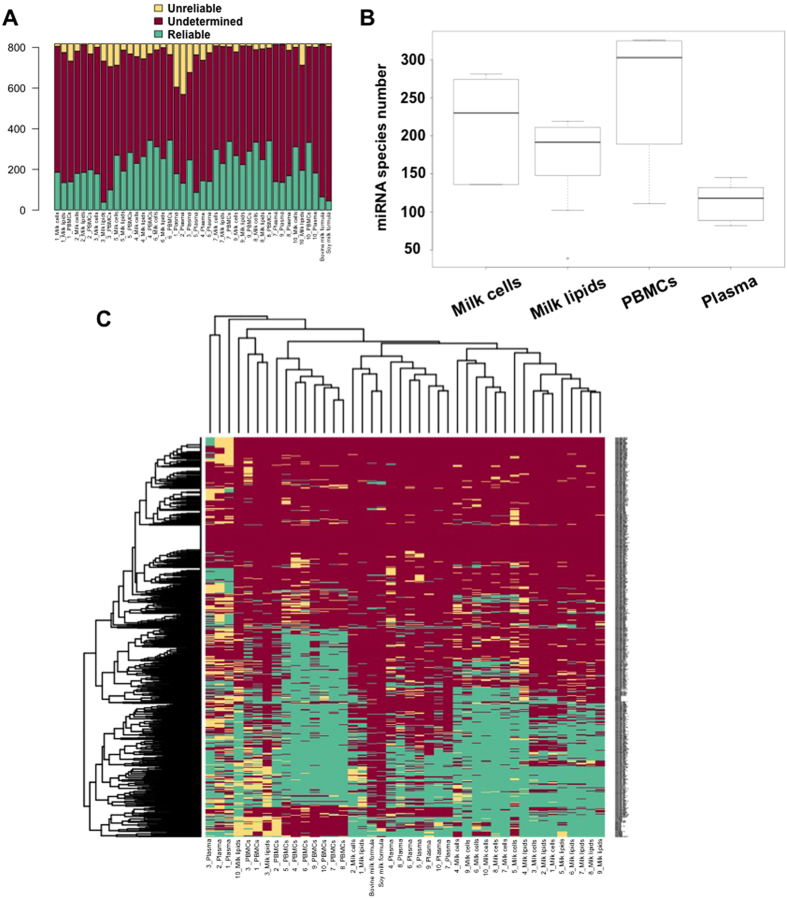
**(A)** Ranking for the classified Ct values across each sample run on the OpenArray platform. Reliable miRNAs had 8 ≤ Ct ≤ 29 and were detected in at least 4 samples per group, whilst unreliable miRNAs had markedly low Ct values (<5) or showed excessive variation between replicates. Undetermined miRNAs correspond to a failed assay or a rare miRNA species (Ct > 29). **(B)** Boxplot showing the number of miRNA species in all mothers and each sample group (n = 10 per sample group). **(C)** Heat map showing an overview of the Ct quality categories that have been assigned to the different miRNAs. The categories correspond to “Reliable”, “Undetermined” and “Unreliable” with the colour codes “green”, “red” and “yellow”, respectively.

To investigate the miRNA composition of HM and maternal blood, TaqMan OpenArray was used to measure Ct values for 681 human known mature miRNAs. Using a first criterion of 8 ≤ Ct ≤ 29, HM cells and fat, and PBMCs and plasma were found to conserve 450, 337, 488, and 319 miRNAs, respectively. To determine the most reliably measured miRNAs, we used a novelty criterion stating 8 ≤ Ct ≤ 29 and miRNA presence in ≥4 out of the 10 samples tested for each of milk cells, milk fat, PBMCs and plasma. Unreliable miRNAs were determined based on Ct < 5 or excessive variation between replicates. Undermined miRNAs (miRNAs that were tested in the OpenArray panel but were not detected in any of the samples examined) did not match the above criteria (Fig. 2A). Using this novelty criterion, 292 miRNA species were determined in HM cells, 242 miRNAs in HM fat, 345 miRNAs in PBMCs, and 219 miRNAs in blood plasma (Table 1, Supplementary Table S1A). Plasma was diverse in miRNA composition, with great variation between participants, and contained fewer miRNA species than PBMCs and HM cells (*p* < 0.001) or HM fat (*p* = 0.015). HM cells contained more miRNA species than HM fat (*p* = 0.038). PBMCs conserved a high number of miRNAs in most samples, which was significantly higher than in HM fat (*p* = 0.001), but not statistically different to HM cells (*p* = 0.084) (Fig. 2B). Of the miRNA identified, the top 10 and 20 most highly expressed in all four sample groups (HM cells and fat, and maternal PBMCs and plasma) are listed in Table 1 and Supplementary Table S2, respectively. All the miRNA species detected in each sample are listed in Supplementary Table S1B.

**Figure 3:**
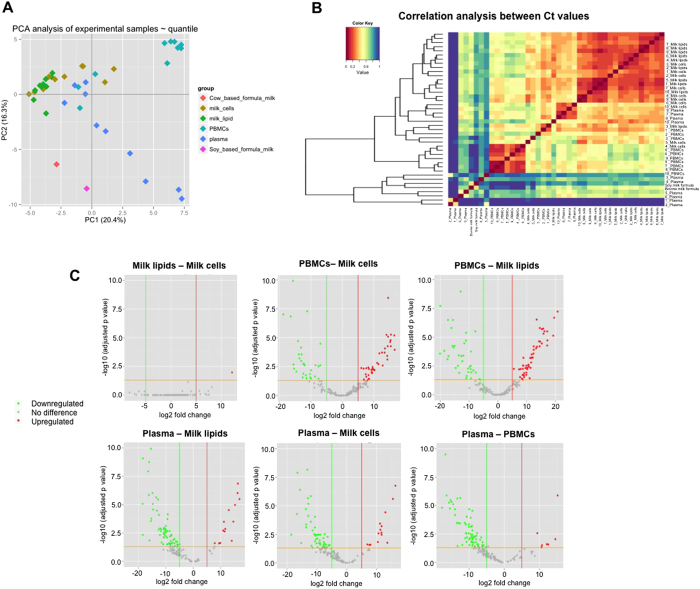
**(A)** Principal Component Analysis (PCA) showing the distribution and clustering of the individual sample groups. The miRNA content in PBMCs form a tight cluster mainly in the top right of the graph, whilst the plasma samples show a broader distribution suggestive of high inter-individual biological variation. The HM cell and fat miRNAs are clustered together and share a similar space on the graph. **(B)** Correlation plot demonstrating the relationship between different sample groups. The regions of the graph that are shaded in hotter colours correspond to more similar profiles of miRNA presence and relative abundance. The HM cell and fat samples are all well correlated with each other, whilst a slight correlation between maternal PBMCs and plasma samples could be seen. Two plasma samples were markedly different to all other samples. **(C)** Differential expression analysis for the reliable miRNAs identified using Linear Models for Microarray Analysis (limma) in the R HTqPCR package. Results are presented in volcano plots, where the log-fold change is plotted on the x-axis and the −log10 (adjusted *p* value) on the y-axis. The six volcano plots demonstrate a comparison between the four sample groups (HM cells and fat, and maternal PBMCs and plasma) by graphic fold change versus significant (*p* < 0.05) to exhibit differences in miRNA expression between sample groups.

Heat map analysis and plotting of miRNA profiles showed that most miRNAs in HM cell and fat samples are clustered together, whilst PBMC and plasma miRNAs do not significantly relate to any of HM cells or fat samples (Fig. 2C). The relationship between sample groups was further examined using Principal Component Analysis (PCA), which demonstrated the distribution and clustering of the individual group samples (Fig. 3A). PBMCs formed a tight separate cluster, whilst plasma miRNA showed a broader distribution suggestive of biological variation. Most of the HM cells and fat samples were grouped together.

Differential miRNA expression analysis for the reliable miRNAs identified was performed in R (HTqPCR package) using Linear Models for Microarray Analysis (limma) (Fig. 3C). The full set of miRNAs and their differential expression *p* values are shown in Supplementary Tables S3–S13. HM cell and fat miRNA expression was highly correlated, with the exception of hsa-miR-564, which was higher in fat than in milk cells. Most of the detected miRNAs in the other sample groups were differentially expressed amongst them. These findings were confirmed using correlation analysis in DataAssist, which showed a high correlation in miRNA expression between HM cells and fat (r = 0.90), whilst the correlation between PBMC miRNAs and miRNAs in HM cells or fat was poorer (r = 0.62; r = 0.57, respectively). Plasma miRNAs were expressed at distinctly different levels than all other three sample groups (r = −0.08, −0.001, −0.27 for HM cell, fat, and PBMCs comparisons respectively).

**Figure 4:**
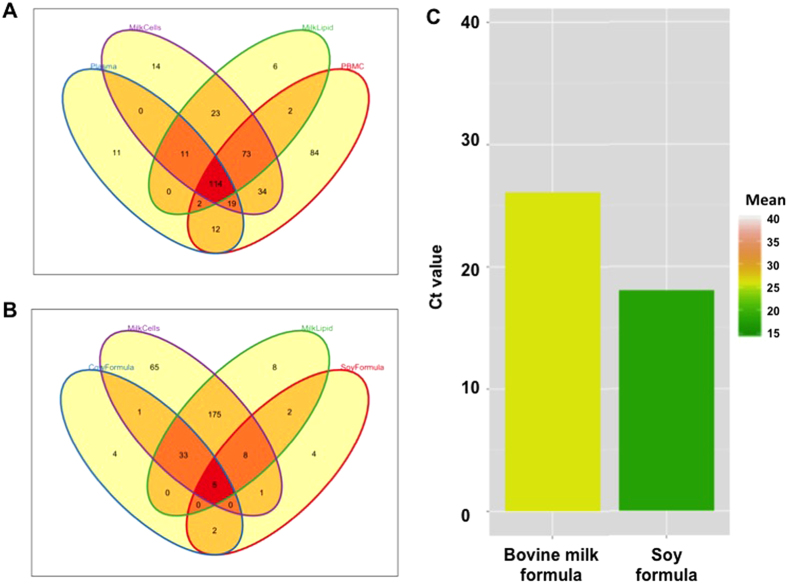
Shared reliable miRNAs (8 ≤ Ct ≤ 29 and present in at least 4 samples per group) between the four sample groups examined. The bovine milk- and soy-based formulae are the results of a single assay and the observations are only illustrative (8 ≤ Ct ≤ 35). **(A)** Euler diagram showing overlapping reliable miRNA species between sample groups. **(B)** Euler diagram showing the number of reliable miRNA species in the HM cell and fat samples and their overlap with infant formulae. **(C)** Box plot showing high expression of plant-based miR-159a (16 replicates in each infant formula) in the two formulae tested.

Further, a comparative analysis of the miRNA species present in each group was performed using the most reliable miRNA identified. This also confirmed the high correlation in miRNA content between HM cells and fat (Fig. 3B). PBMC miRNAs were weakly related to HM miRNAs and also to plasma miRNAs (Fig. 3B). Euler diagrammatic analysis revealed the overlapping (most reliable) miRNAs between the different sample groups examined, showing 43 miRNAs that were specific to HM and were not found in maternal blood (Fig. 4A, Supplementary Table S14A). Of these, 14 miRNA were specific to HM cells, 6 were specific to HM fat, and 23 were commonly specific between HM cells and fat. Similarly, 84 miRNA species were specific to maternal PBMCs, and 11 to plasma (Fig. 4A, Supplementary Table S14A). In total, 221 miRNA species were shared between HM cells and fat (Fig. 4A, Supplementary Table S14A). Of the 681 miRNAs assayed, 114 were detected in all four sample groups, 34 were specific to HM cells and PBMCs, 2 were specific to HM fat and PBMCs, and 12 were specific to PBMCs and plasma (Fig. 4A, Supplementary Table S14A). Interestingly, no miRNAs were identified that were specific to plasma and HM only. These data provide evidence supporting the endogenous synthesis of the majority of HM miRNA in the lactating mammary epithelium, with a small contribution of the maternal circulation. A proportion of HM miRNA appear to be unique to lactation, as they were not detected in maternal blood.

### Infant formulae are low in human miRNA compared to human milk

Both infant formulae examined (bovine milk-based and soy-based) contained very few human mature miRNA species, and those were present at very low expression levels compared to HM fractions (Fig. 2A). Although the RNA input of the soy-based formula was higher than that of the bovine milk-based formula (Table 2), the latter contained 45 mature human miRNAs compared to 22 in the soy-based formula with 8 ≤ Ct ≤ 35, whilst only 26 and 19 respectively with the more reliable 8 ≤ Ct ≤ 29 (Supplementary Table S15). These differences between the two formulae potentially reflect differences between animal and plant miRNA. The miRNA content and expression patterns of the bovine milk-based infant formula were more similar to that of HM than the maternal PBMCs or plasma, and less so for the plant-based formula (Fig. 3B). Of the miRNA detected in the formulae, 33 were common between the bovine milk-based formula and HM cells and fat, whilst only 8 miRNAs were common between the soy-based formula and HM fractions (Fig. 4B, Supplementary Table S14B). Interestingly, miR159a, which is known to be plant-specific^35^ and was not expressed in any of the HM samples, was detected in not only the soy-based formula, but also in the bovine milk-based formula, at 16 replicates and high expression levels compared to the other identified miRNAs in this formula (Fig. 4C, Supplementary Table S16).

### Pathway enrichment and ingenuity pathway analyses reveal a plethora of biological functions associated with human milk miRNAs

**Figure 5:**
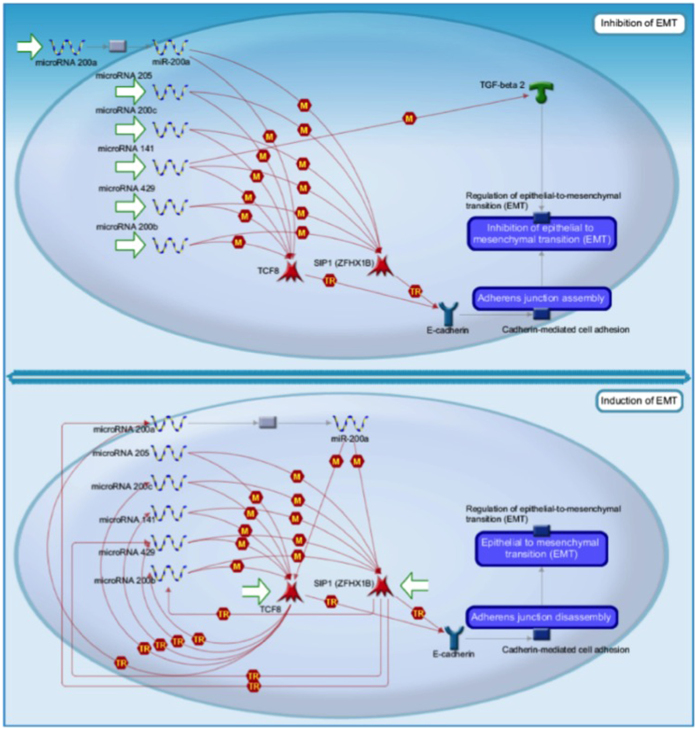
Example of molecular pathway (epithelial-to-mesenchymal transition, EMT) controlled by some of the most highly expressed reliable miRNAs detected in HM (miR-200a/b/c/, miR-429, miR-141, and 205), and how they interact with other genes to regulate EMT.

Pathway enrichment analysis of some of the significantly differentially expressed miRNAs in HM cells and fat (miR-200a; 205; 200c; 141; 429; 200b; 106b; 20a; 17; 34a; 34c; 340–5p; 137–3p; 195) was done using the MetaCore pathway analysis tool pipelines by GeneGO (GO)^36^. Several of these miRNAs were found to interact in the molecular pathways regulating the inflammation action of Endothelin-1, cardiovascular disease, and sickle cell disease. They are also known to be involved in microphthalmia-associated transcription factor (MITF) in melanoma, epithelial-mesenchymal transition (EMT), lung epithelial progenitor cell differentiation, and tumor protein p53 signaling in prostate cancer (Fig. 5, Supplementary Figures S2–S5).

Due to the limited number of miRNA that are recognized and can be analyzed using MetaCore, we also employed ingenuity pathway analysis to further explore functional and disease pathways associated with the reliably expressed miRNAs (8 ≤ Ct ≤ 29) across all samples through 6 mir seed regions, which is the mRNA-miRNA binding site. All the identified miRNAs were mapped to biological processes to determine their contribution in a variety of normal and abnormal conditions (Supplementary Tables S17–S21). Due to the overrepresentation of knowledge associated with some diseases including cancer and inflammation, compared to normal biological functions, many miRNAs in this study have been identified to be associated with abnormal conditions. However, numerous miRNAs also have important roles in normal conditions. As shown by the differential expression analysis (Supplementary Table S6), miR-564 was differentially expressed between HM cells and fat, where it was upregulated in HM fat. This miRNA is known to regulate the differentiation of adipose tissue-derived stem cells (ADSCs)^37^, and in the case of HM, it may participate in milk or fat synthesis in the lactocyte. A plethora of biological functions across all sample groups examined were regulated by the reliably identified miRNAs. These functions included cellular development and movement, cell cycle, growth and proliferation, and immune responses (Supplementary Tables S17–S21).

## Discussion

The discovery of novel bioactive components in HM, such as stem cells and miRNAs, re-emphasizes its importance as a nutritional, developmental and protective agent for infants. Increasing evidence suggests the involvement of miRNAs in both normal mammary development and function and infant health via HM^12^,^28^,^38^. The abundance of miRNA in milk and their stability in the GI tract in adults further support their survival and functional significance in the breastfed infant^12^,^13^,^25^,^28^. Over the past few years, HM miRNAs have been investigated in either skim milk^13^,^14^,^28^ or milk fat and exosomes^25^, whilst the cellular component of milk has been largely ignored despite its high RNA content^33^. We used the OpenArray Taqman technology to profile 681 human mature miRNAs in the cell and fat fractions of HM and compare it with maternal blood (PBMCs and plasma) as well as with two commercially available bovine milk- and plant-based formulae. HM cells were found to conserve higher quantities of miRNA, both in total content and miRNA species composition, compared to previously studied HM fat and skim milk^13^,^14^,^25^. The miRNA composition of the cell and fat fractions of HM was similar, whilst maternal PBMCs and plasma displayed distinctly different miRNA profiles to HM. This finding, together with the previously shown dominance of lactocytes in mature HM of healthy mothers and infants^7^,^8^,^39^, suggest endogenous synthesis of the majority of HM miRNA in the mammary epithelium, with a small contribution of the maternal circulation.

The total RNA content enriched in miRNA was not significantly different between PBMCs and HM cells (Fig. 1E,F), indicating similar transcription activity in both milk-derived mammary epithelial cells and maternal blood-derived PBMCs. In contract, HM fat conserved higher RNA concentration compared to maternal plasma (Fig. 1B), with similar miRNA content to that of HM cells. This is in accordance with the origin of milk fat globule from the lactocyte^7^ and with previous milk fat miRNA studies^25^. Thus, HM fat, in addition to being an essential nutritive component for the infant^40^, is also an important carrier of bioactive miRNA molecules to infants. No relationship was seen between HM fat content and the total RNA of HM fat, which is in agreement with the fact that total HM fat content does not necessarily reflect the number of fat globules^41^. This suggests that the fat miRNAs are primarily packaged within the fat globule or other microvesicles such as exosomes contained within the milk fat. A positive relationship between HM cells or PBMCs and total RNA content was seen as expected. However, this was stronger for PBMCs, which represent a specific cell type in contrast to HM cells, which are a heterogeneous population of cells including primarily lactocytes (in mature milk from healthy mothers and infants), but also smaller populations of stem cells, progenitor cells, and immune cells^7^,^8^,^9^,^39^ .The weaker association between HM cells and total RNA content may reflect differing RNA transcription between these different cell populations of HM.

Although both HM cells and PBMCs were rich in different miRNA species (292 and 345 miRNAs, respectively) and not significantly different to each other in our study cohort (*p* = 0.084), HM fat had a lower miRNA species number (242 miRNAs) (*p* = 0.038 for the comparison with HM cells; *p* < 0.001 for the comparison with PBMCs), followed by maternal plasma (219 miRNAs) (*p* < 0.001 for the comparison with HM cells and PBMCs; *p* = 0.015 for the comparison with HM fat). The poor miRNA species content of maternal plasma has also been seen in other body fluids, such as human urine^14^, and also in skim milk^13^. Therefore, the HM cellular component conserves more miRNA species than the HM fat and skim milk fractions, emphasizing the need to include it in milk miRNA investigations, particularly since it better represents what the infant receives. However, caution needs to be exercised when analyzing whole milk samples to ensure complete lysis of HM cells and membranous microvesicles for miRNA extraction. This is better performed when fractionating the HM into cells, skim milk, and fat immediately upon expression and prior to freezing, and then extracting miRNA after rigorous cell lysing, as has been previously described^34^,^42^.

Differential miRNA expression analysis together with heat mapping and comparative analysis of miRNA species showed strong similarities in miRNA composition and profiles, including expression levels, between HM cell and fat fractions. At the same time, maternal blood, both its PBMCs and plasma, had distinctly different miRNA profiles to HM cells and fat (Fig. 3A–C). It has been previously shown that in mature HM of healthy mother-infant dyads, which is what was analysed in our study, the dominant cell type is the lactocyte (secretory mammary epithelial cell), which also secrets the HM fat^7^,^8^,^39^. Therefore, our findings strongly support the origin of HM cell and fat miRNAs primarily from the mammary epithelium via endogenous synthesis in the lactocytes. The maternal circulation may still likely have a small contribution to milk miRNA. This is in agreement with a recent study in the tammar wallaby reporting a weak correlation between maternal serum and skim milk miRNAs, suggesting that milk miRNAs are primarily synthesized in the mammary gland^24^.

The origin of HM miRNA primarily from the mammary gland emphasizes their potential use as biomarkers of both lactation performance and the health of the gland. Indeed, HM immune cells have been shown to rapidly respond to maternal infections, with the most pronounced responses seen in abnormal conditions of the lactating breast such as mastitis^8^,^39^, which if left untreated or are managed late, can result in early cessation of breastfeeding, with detrimental effects to both the infant and the mother^43^. Recent studies have shown distinct miRNA responses in the milk of the dairy cow during mastitis, consistent with the immune cell response^44^. Moreover, family members of miR-29 (miR-29a/b/c), an abundant miRNA in mammalian milk, which was also found to be highly expressed in HM cell and fat fractions in our study, was shown to epigenetically regulate lactation performance in the dairy cow^45^. The distinct changes of milk miRNA in response to the status of the mammary gland together with our data supporting the mammary origin of milk miRNA highlight their potential diagnostic value as non-invasive and easily accessible biomarkers of mammary gland function and health to facilitate timely management of lactation difficulties and maintenance of breastfeeding for longer periods. In a similar context, circulating miRNA in plasma have been successfully used as early biomarkers^46^,^47^ of aberrant growth in breast cancer^48^ and of other diseases such as type 2 diabetes^49^.

To give insight into the content of artificial infant formulae in miRNA, we compared the miRNA profiles of a bovine milk-based and a soy-based formulae that are in high demand in the Australian market. We found very few human mature miRNAs in both of these formulae and a poor miRNA representation compared to HM (Fig. 2A). The bovine milk-based formula clustered more closely with HM cell and fat miRNAs than the soy-based formula (Fig. 2A,B), likely due to the mammalian milk basis of the former versus the plant basis of the latter, yet still very poorly correlated with HM. Skim bovine milk has been shown to harbor 245 miRNAs^22^, some of which are similar to those in HM, but most of these miRNAs were not found to be present in the bovine milk-based formula analysed in our study. Although miRNAs have been shown to be highly stable in infant formula^22^,^50^, the first step of the formula manufacturing process (for both animal milk-based and plant-based formulae) discards the milk fat layer as well as cell debris by extremely high speed centrifugation followed by pasteurisation^51^,^52^, and therefore likely excludes the sources that are rich in miRNA.

Interestingly, miR-159a was the most highly expressed miRNA in both the bovine milk-based and the soy-based formulae (Fig. 4C). This is a plant-specific miRNA not detected in any of the HM samples analyzed. It is possible that it originated from the nutrition of the animals from which milk was sourced for the bovine formula. Moreover, it may come from vegetable fats, such as soybean oil, that are added to the bovine formula during manufacturing as per its ingredients, though intentional addition of this miRNA or contamination during preparation and processing cannot be excluded either. This requires validation with further samples of this formula. It is not known whether this miRNA survives in and can be absorbed by the GI tract of the infant to exert gene regulatory functions, and this warrants further investigation. This is likely given that other plant food-derived miRNAs, such as miR-168a, have been shown to influence gene expression in adult humans^20^.

Recently, exogenous miRNAs have been experimentally proven to regulate gene expression in mammalian cells^20^,^30^,^31^. These miRNAs are transferred to humans via consumption of food, therefore it is highly likely that the same transfer of HM miRNA to the breastfed infant occurs, especially since the neonatal stomach is less acidic^53^ and the gut highly leaky early in life^54^. A recent study in adult humans demonstrated that after 4 to 8 hours of consuming bovine milk, miR-29b and miR-200c increased in the plasma, returning to baseline levels after 24 hours of the initial consumption^30^. Further, after bovine milk consumption, the expression of runt-related transcription factor 2 (RUNX2), targeted by miR-29b, was elevated in PBMCs^30^. A recent study by Arntz *et al.* further demonstrated uptake of milk-derived miRNA by mammalian cells and a therapeutic function in ameliorating experimental arthritis in mice^31^. Therefore, the high presence of miRNAs in HM further supports its function as a biofluid initiating epigenetic signals in infants that could potentially influence infant development and health. In our study, GeneGo analysis and Ingenuity Pathway Analysis (IPA) revealed a number of biological functions and pathways that are controlled by miRNA enriched in HM, including immunity, growth and development, cell proliferation and apoptosis, lung epithelial progenitor cell differentiation, and epithelial-to-mesenchymal transition (EMT) (Fig. 5). EMT has been found to be a key player in cell differentiation, motility and migration in multiple tissues and organs, particularly during embryogenesis and in cancer^55^. It has been shown to be normally present in both the human^33^ and the murine mammary gland^56^, with potential important functions in normal breast remodeling required for milk synthesis. Our results suggest that the EMT process in the normal lactating breast may be mediated by pregnancy- and lactation-specific miRNAs, which requires further investigation.

Consistent with previous studies in HM, some of the most abundant milk miRNAs detected here are known to contribute to metabolic processes, decrease the cancer risk of infants^25^, participate in the development of the infant’s immune system, and protect infants from infections^13^,^25^,^28^. Moreover, miRpath^57^ and KEGG^58^ analyses showed that the majority of the top 10 highly expressed miRNAs in both HM fractions examined here (cells and fat) are involved in the regulation of the cell cycle and the RNA transportation process during development (Supplementary Figure S1A). Normal cell cycle and RNA transport in the lactating breast is required for normal cell differentiation and proliferation of the lactocyte^7^, and also to prevent breast cancer initiation by controlling cell proliferation^59^. In contrast, the most highly expressed miRNAs in PBMCs were found to be involved in different molecular pathways to those of HM, such as the maintenance of normal gene activity of K-Ras, HER2 and CDK4, which are known oncogenes^60^ (Supplementary Figure S1B). Further, miRNAs found to be highly expressed in the maternal plasma are known to be involved in the control of oncogenes in melanoma, pancreatic and colorectal cancers^58^.

Collectively, our data together with previous studies demonstrate that miRNAs are abundant in HM and likely play significant roles in the development and normal function of the lactating mammary gland, and in the HM fed infant. We have provided evidence that HM miRNA are primarily synthesized in the mammary epithelium, and may therefore be used as novel diagnostic biomarkers of lactation performance and breast infection. Further research is required to identify the functions of these miRNAs and examine potential novel miRNAs that may be present in the milk and the breast. Moreover, factors that may influence the miRNA content and expression levels of HM should be investigated in an effort to standardize milk miRNA studies and elucidate the maternal-infant interaction in the regulation of these molecules during breastfeeding.

## Materials and Methods

### Ethics, sample collection and processing

This study was approved by the Human Research Ethics Committee of The University of Western Australia, and the methods were carried out in accordance with the approved guidelines. Informed written consent was provided by all participants, which included 10 exclusively breastfeeding dyads in month 2 postpartum (week 4–8) to ensure established lactation. All participating dyads and their infants were healthy at the time of collection. The workflow from sample collection to analysis is shown in Supplementary Figure S6. Fresh HM samples (24–78 mL) were collected early in the morning. Aseptic collection of the samples was carried out using a breast pump, sterile bottles and other accessories (Medela AG, Switzerland). Maternal blood samples were collected at the time of milk collection by an accredited phlebotomist. All the blood samples were collected into EDTA tubes (Becton Dickinson, Mountain View, CA, USA). Samples were transferred immediately to the laboratory in the dark for processing. HM samples were processed for miRNA analyses as previously described^33^,^34^. Briefly, fresh milk samples were diluted 1:1 with phosphate buffered saline (PBS; Gibco, Life Technologies, Foster, CA), and were then centrifuged at 800 *g* for 20 min at 20 °C for fractionation. HM cells and fat were transferred separately into new RNAse free tubes. Cells were then washed three times with PBS, stained with Trypan blue (ProSciTech, Queensland, Australia) and counted using a haemocytometer as previously described^33^. Fat samples were centrifuged twice at 450 *g* for 20 min at 20 °C to obtain a pure fat fraction. For blood fractionation, the whole blood samples were collected in EDTA-coated centrifuge tubes and were centrifuged at 800 *g* at 20 °C for 10 min to separate the plasma from cells. The plasma was then transferred to a new tube and centrifuged further at 3,500 *g* for 20 min at 4 °C to remove all residual cells and other debris. Blood peripheral mononuclear cells (PBMCs) were isolated from blood cell samples based on Secoll separation (Serana, Australia). Briefly, after transferring the plasma into a new tube, the buffy coat in the top layer of the whole blood was transferred to a new tube with PBS. PBMCs in PBS were gently overlaid onto 4 mL of Secoll in a new tube and centrifuged at 800 *g* for 15 min at 20 °C. The middle layer containing PBMCs was collected and was washed three times in PBS, then counted as described for HM cells above. Plasma samples were centrifuged for 15 min at 4 °C at 14,000 *g* to further purify plasma. miRNAs were extracted from all samples immediately without cryopreservation.

### Infant milk formulae

Equal amounts (2 g) of two different types of infant formula powder were dissolved in 4 mL of Trizol LS Reagent (Invitrogen, CA, USA). Standard, whey dominant, infant formula known as bovine milk-based formula (S-26 Gold) and soy-based infant formula (S-26 Gold Soy) manufactured by Aspen Nutritional Australia, were used. As per the manufacturer’s bottle instructions, both are considered to be suitable for infants from birth to one year old. The semi-dissolved powder in Trizol was incubated for 30 min at 37 °C for complete dissolution.

### Milk fat content

Fat content of whole fresh HM samples was measured using Creamatocrit Plus (Medela, Inc, McHenry, Illinois) as previously described^61^. Briefly, whole HM was taken up by a capillary tube, then centrifuged for 10 min at 11,731 *g* in a microcentrifuge (BHG Hermle, Germany) to separate the milk fat from skim milk and cells.

### Extraction and quantification of miRNA

miRNA were isolated from different fractions of HM according to our previous study^34^, where the miRNeasy mini kit (Qiagen, Hilden, Germany) was used to extract miRNAs for HM cells and maternal PBMCs, the miRCURY RNA Isolation-Biofluids Kit (Exiqon, Vedbaek, Denmark) for HM fat and both infant formulae, and the mirVana PARIS Kit (Ambion, Austin, TX, USA) for maternal plasma. These three miRNA extraction kits all use filter column-based methods. All the extractions were done according to the manufacturer’s protocol and as described previously ensuring complete lysis of cells and membranous components via^34^. The volume of milk fat and lysis reagent for the miRCURY RNA Isolation-Biofluids Kit were increased to 400 μL and 120 μL, respectively. The concentration and purity of the extracted miRNA were measured using a NanoDrop 2000 Spectrophotometer (Wilmington, DE, USA) and Agilent Bioanalyzer 2100 (Agilent, CA, USA) with the RNA 6000 NanoChip kit. All miRNA samples were then stored at −80 °C until further analyses incubated for 30 min at 37 °C for complete dissolution.

### Reverse transcription (RT), preamplification and TaqMan OpenArray analysis

The expression levels of 681 human mature miRNAs that have been functionally validated with miRNA artificial templates were profiled in the milk, formulae and maternal PBMCs and plasma samples using the TaqMan miRNA OpenArray panel system (Life Technologies, CA, USA). The panel originally profiled 758 human mature miRNAs, however since its release, recent updates of miRNA integrity and function confirmed 681 out of the 758 targets as true human miRNAs (Supplementary Table S22). Human ath-miR159a was used as a negative control for the human samples, and RNU48, RNU44 and U6 rRNA were used as housekeeping controls. According to the manufacturer’s instructions, total extracted miRNA (>50 ng of total RNA) was combined with reverse transcription primers using the Megaplex RT Primers (Life Technologies) in a MicroAmp Fast Optical 96 well plate (Life Technologies) to synthesize single strand cDNA. This was followed by preamplification, which employed the Megaplex PreAmp Primers (Life Technologies) to increase the quantity of desired cDNA. All preamplification samples were diluted (1 in 40) using 0.1X TE pH 8.0 (Promega, WI, USA). TaqMan OpenArray Real Time PCR Master Mix was added to the diluted samples to amplify the preamplified cDNA using 681 unique human mature miRNA primers on the OpenArray 384 well plate. The samples were loaded from a 384-well plate into the TaqMan OpenArray Panel using the OpenArray AccuFill System, then the panel was analysed on the TaqMan OpenArray Cycler platform (Life Technologies). Cycle threshold (Ct) values for targeted mature miRNAs, including the controls, were automatically measured and calculated using the supplied OpenArray software. Infant formulae made from soy milk expectedly contained ath-miR159a, which is a plant-specific miRNA. Therefore, ath-miR159a was used as a negative control for HM and blood samples, and as an endogenous target miRNA for both infant formulae samples. This miRNA (**5**′UUUGGAUUGAAGGGAGCUCUA3′; miRBase accession MIPF0000010) was screened for 16 replicates in each formula, and was considered a miRNA with high reliability.

### Gene Ontology and gene target analysis

Pathway enrichment analysis of the reliable (8 ≤ Ct ≤ 29 and detected in at least 4 samples per group) and significantly differentially expressed miRNAs for each comparison was done using the MetaCore pathway analysis tool by GeneGO (Thomson Reuters). The software only recognized and analyzed miR-200a; 205; 200c; 141; 429; 200b; 106b; 20a; 17; 34a; 34c; 340–5p; 137–3p; 195. *p* values were calculated using the hypergeometric test^62^ and adjusted for multiple testing using the False Discovery Rate “FDR” method^63^. GeneGO categories were found to be significant (adjusted p value <0.05) for each list of differentially expressed miRNAs, and pathways were generated. Due to the limited number of miRNA that are recognized and can be analyzed using MetaCore, Ingenuity Pathway Analysis was also employed to map all the reliable miRNAs to IPA database to identify associated biological or pathological functions.

### Statistical analyses

The qRT-PCR quality characteristics, the differential expression analysis, and comparative analysis for each of the samples were investigated using the R Studio Version 0.98.1103 package^64^ and the HTqPCR software, which provides biostatistical tools for the analysis of Ct values obtained from the TaqMan OpenArray assays^65^, including data loading, quality assessment, normalisation, visualisation and parametric or non-parametric testing for statistical significance in Ct values between features (the individual miRNAs). Linear mixed effects (LME) models, general linear hypothesis tests, and graphical exploration of the data were used to examine associations between cell and total RNA enriched in miRNA content. Tukey’s HSD test was employed to identify differences in miRNA species between different sample groups (HM cells and fat, and maternal PBMCs and plasma). P < 0.05 was considered statistically significant. Reliable miRNA were 8 ≤ Ct ≤ 29 and detected in at least 4 samples per group, whilst unreliable miRNA correspond to markedly low Ct value (<5) or showing excessive variation between replicates. Undetermined miRNA correspond to a failed assay or a rare miRNA species (Ct > 29). The DataAssist software (Life Technologies, Foster, CA) was used to calculate the delta Ct value and fold change of each identified miRNA and to perform the differential expression analyses to confirm the results obtained by the HTqPCR.

## Additional Information

**Accession code**: The data set of this study is available in GEO database under accession number GSE66358.

**How to cite this article**: Alsaweed, M. *et al.* Human milk miRNAs primarily originate from the mammary gland resulting in unique miRNA profiles of fractionated milk. *Sci. Rep.* **6**, 20680; doi: 10.1038/srep20680 (2016).

## Supplementary Material

Supplementary Tables

Supplementary Figures

## Figures and Tables

**Table 1:** Sample characteristics for HM cells and fat, and maternal PBMCs and plasma (n = 10 for each sample group), including quantity and quality of total RNA enriched in miRNA extracted from using NanoDrop 2000 and Bioanalyzer 2100, total number of miRNA species detected, and top 10 most highly expressed miRNAs.

Extraction kit used	Milk cells	Milk fat	PBMCs	Plasma
	miRNeasy mini	miRCURY Biofluids	miRNeasy mini	mirVana
Cell content/mL milk or blood (mean ± SD) (cell viability %)	435,787 ± 311,967 (92.5)	–	724,073 ± 309,449 (85.1)	–
Fat content of milk (%) to (mean ± SD)	–	8.4 ± 3.0	–	–
Total RNA (mean ± SD) using NanoDrop 2000^1^	2,525 ng/10^6^ cells ± 1048	29 ng/μl of fat ± 17.5	2,648 ng/10^6^ cells ± 1821	4.62 ng/μl of plasma ± 3.0
RNA purity (OD 260/280) (mean ± SD) using NanoDrop 2000	2.04 ± 0.02	2.00 ± 0.04	2.07 ± 0.01	1.26 ± 0.46
Total RNA (mean ± SD) using Bioanalyser 2100^1^	2,674 ng/10^6^ cells ± 1173	29 ng/μl of fat ± 15.04	2,709 ng/10^6^ cells ± 1821	0.09 ng/μl of plasma ± 0.08
RNA Integrity Number (RIN) (mean ± SD) using Bioanalyser 2100	7.2 ± 0.97	3 ± 1.2	7.5 ± 1.01	1.6 ± 0.82
Number of detectable miRNA species^2^	292	242	345	219
Highly expressed miRNAs	hsa-miR-146b	hsa-miR-200c	hsa-miR-106a	hsa-miR-17
	hsa-miR-200c	hsa-miR-146b	hsa-miR-17	hsa-miR-146a
	hsa-miR-30b	hsa-miR-191	hsa-miR-26a	hsa-miR-16
	hsa-miR-191	hsa-miR-193b	hsa-miR-454	hsa-miR-106a
	hsa-miR-19b	hsa-miR-30b	hsa-miR-20a	hsa-miR-575
	hsa-miR-193b	hsa-miR-19b	hsa-miR-186	hsa-miR-96#
	hsa-miR-223	hsa-miR-16	hsa-miR-29a	mmu-miR-379
	hsa-miR-16	hsa-miR-24	hsa-miR-30b	hsa-miR-30b
	hsa-miR-106a	hsa-miR-30c	hsa-miR-155	hsa-miR-191
	hsa-miR-30a-5p	hsa-miR-106a	hsa-miR-30c	hsa-miR-199a-3p

(SD: standard deviation).

^1^Total RNA (ng) per 1 × 10^6^ of milk cells or PBMCs, or ng/μL of milk fat or blood plasma.

^2^The number of reliable miRNAs species based on our criterion of presence in ≥4 samples per group with 8 ≤ Ct ≤ 29.

**Table 2:** Characteristics of extracted miRNA from bovine milk- and soy-based infant formulae.

	Bovine milk-based formula	Soy-based formula
Total RNA using NanoDrop 2000 (ng/μL of formula)^1^	1.37 ng/μl	116.4 ng/μl
RNA purity (OD 260/280) using NanoDrop 2000	1.33	2.12
Total RNA using Bioanalyser 2100 (ng/μL of formula)^1^	0.1 ng/μl	69.9 ng/μl
RNA Integrity Number (RIN) using Bioanalyser 2100	–	2.2
Number of detectable miRNAs	45	22
Highly expressed miRNAs	hsa-miR-320	ath-miR159a
	hsa-miR-200c	hsa-miR-369-5p
	hsa-miR-574-3p	hsa-miR-151-3p
	hsa-miR-574-3p	hsa-miR-638
	hsa-miR-106a	hsa-miR-601
	hsa-miR-17	hsa-miR-483-5p
	hsa-miR-19b	hsa-miR-155
	hsa-miR-24	hsa-miR-636
	hsa-miR-191	hsa-miR-99b
	ath-miR159a	hsa-miR-520c-3p

Although the criterion of miRNA presence in ≥4 samples with 8 ≤ Ct ≤ 29 cannot be applied in the formula samples tested due to examination of only one sample for each formula, here we report the top 10 most highly expressed miRNAs in each formula, which had 8 ≤ Ct≤ 35. The miRCURY Biofluids kit was used to extract miRNA from both formulae.

^1^2 grams of formula powder dissolved in 4 mL of Trizol LS reagent.
