# Supplementary material for: Human milk miRNAs primarily originate from the mammary gland resulting in unique miRNA profiles of fractionated milk
Source: Sci Rep. 2016 Feb 8;6:20680. doi: 10.1038/srep20680 (PMC4745068; doi:10.1038/srep20680)
Supplement: Supplementary Figures [file srep20680-s2.pdf]

SUPPLEMENTARY FIGURES

Human milk miRNAs primarily originate from the mammary gland resulting in unique miRNA profiles of fractionated milk

Mohammed Alsaweed, Ching Tat Lai, Peter E. Hartmann, Donna T. Geddes, Foteini Kakulas

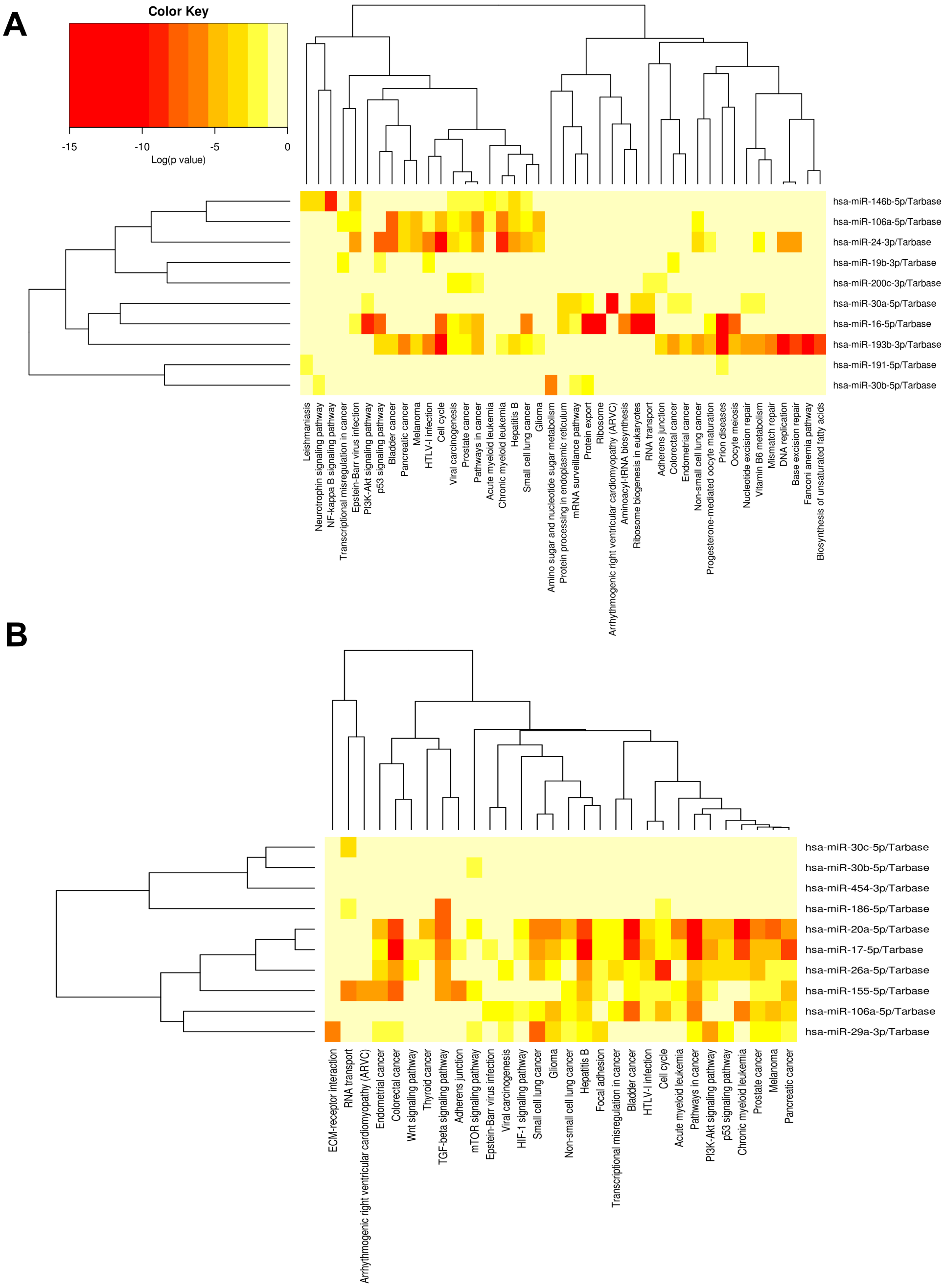

Figure S1. Heat maps showing the top 10 most highly expressed miRNAs in **(A)** human milk cells and lipids and **(B)** maternal PBMCs, and their contribution in different signalling and metabolic pathways annotated to mirPath tool[56].

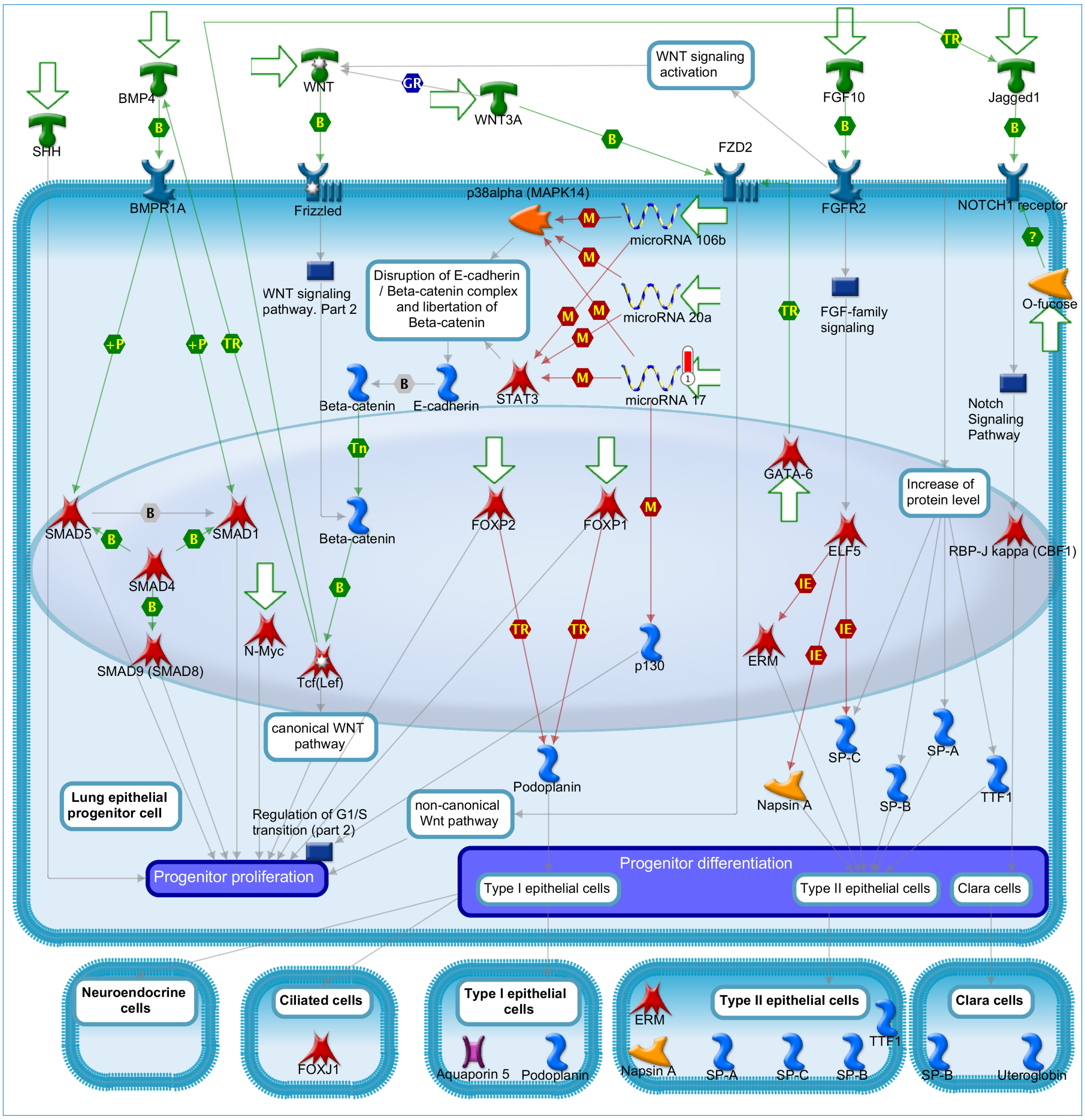

Figure S2. Interaction between and roles of reliable miRNAs that were differentially expressed in the regulation of lung epithelial progenitor cell differentiation (MetaCore pathway analysis).

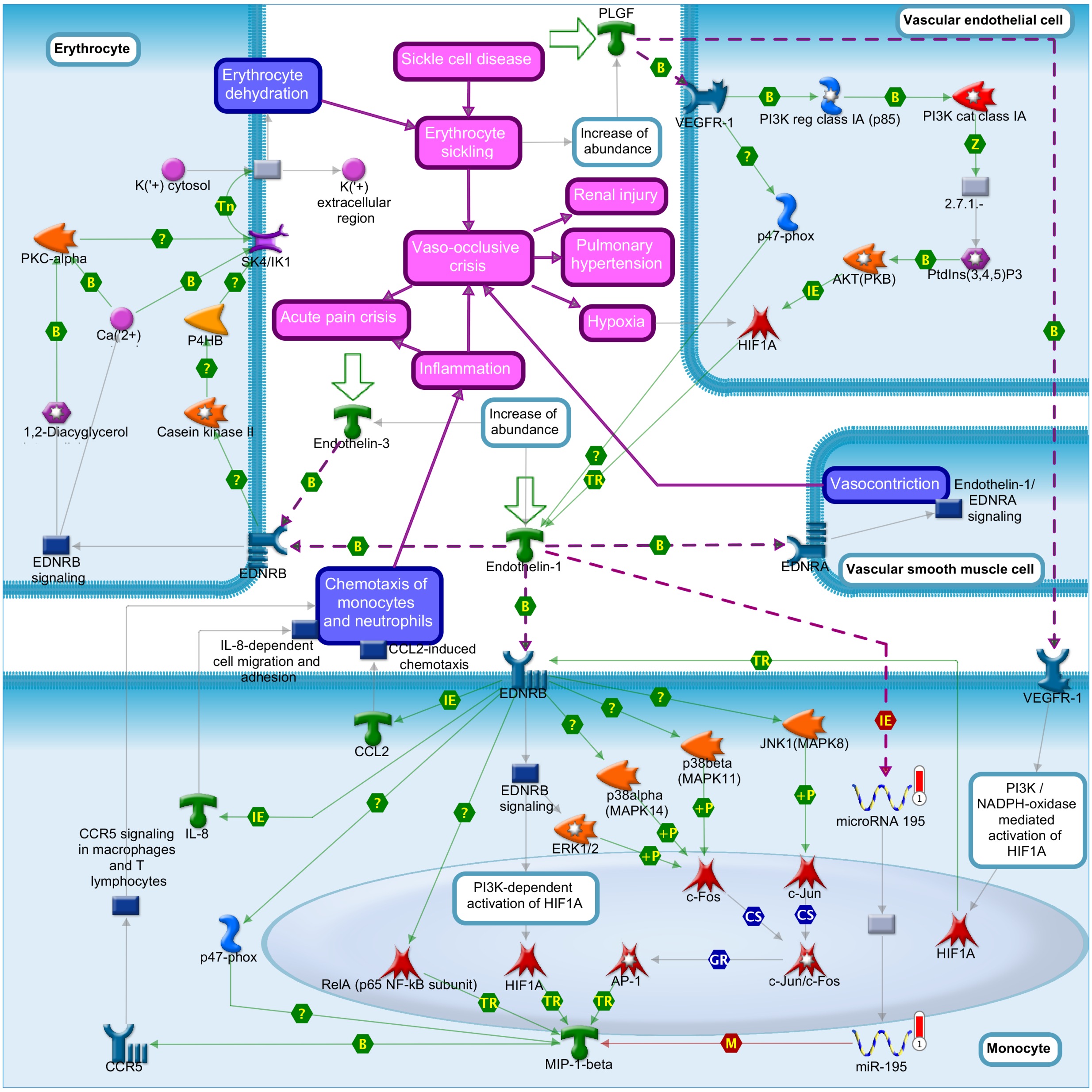

Figure S3. Interaction between and roles of reliable miRNAs that were differentially expressed in endothelin-1 pathway of inflammation and vasoconstriction in Sickle cell disease (MetaCore pathway analysis).

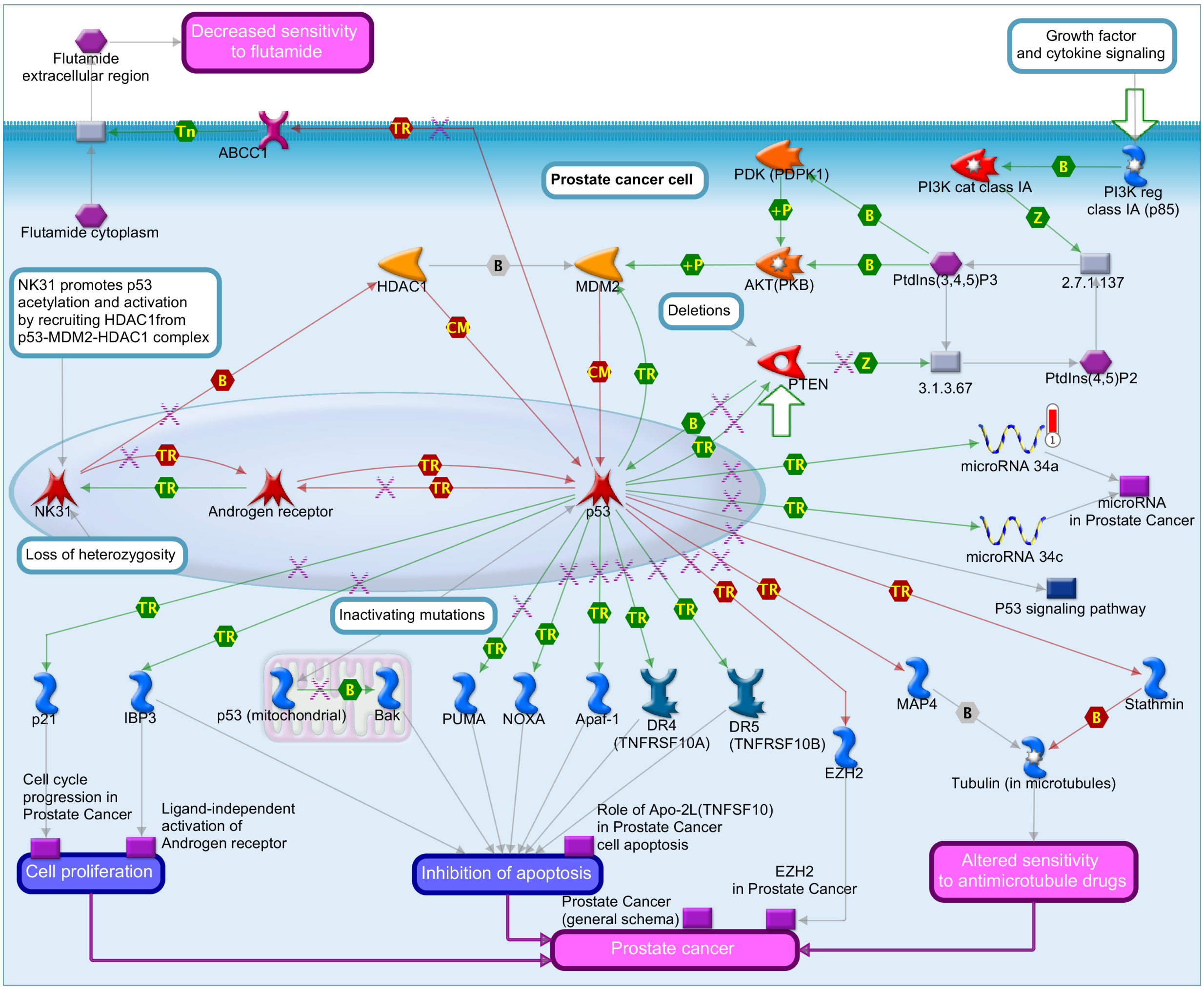

Figure S4. Interaction between and roles of reliable miRNAs that were differentially expressed in p53 signaling in prostate cancer (MetaCore pathway analysis).

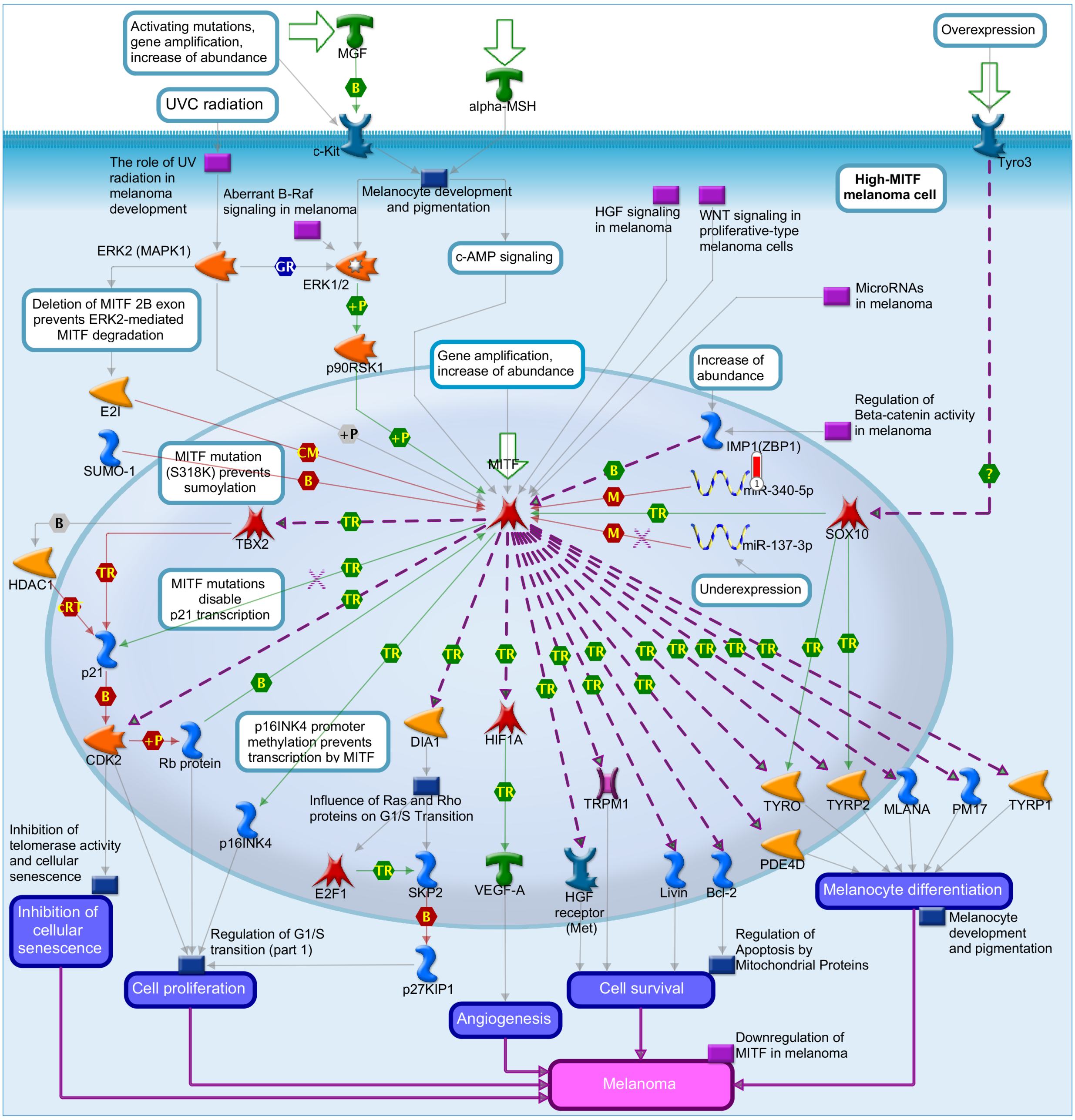

Figure S5. Interaction between and roles of reliable miRNAs that were differentially expressed in the upregulation of MITF in melanoma (MetaCore pathway analysis).

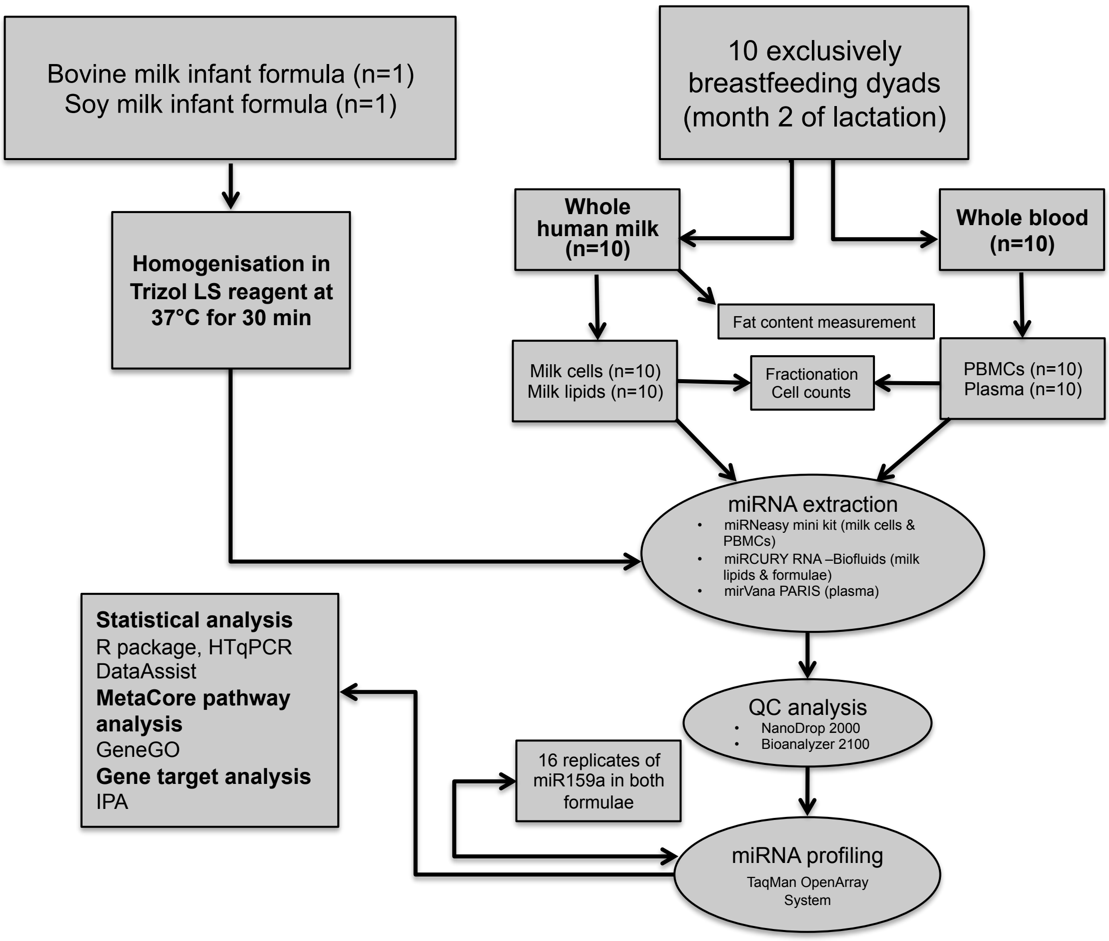

Figure S6. Flowchart describing the main steps of the study.
